# Supplementary material for: Stacking Model‐Based Classifiers for Dealing With Multiple Sets of Noisy Labels
Source: Biom J. 2025 Mar 12;67(2):e70042. doi: 10.1002/bimj.70042 (PMC11898607; doi:10.1002/bimj.70042)
Supplement: Supplementary file 1 — Supporting Information [file BIMJ-67-e70042-s001.zip › density-based-ensemble-model-main/competitors_real_data/netlab-master/netlab-master/help/hesschek.htm]

Netlab Reference Manual hesschek


# hesschek

## Purpose

Use central differences to confirm correct evaluation of Hessian matrix.

## Synopsis

```
hesschek(net, x, t)
h = hesschek(net, x, t)
```

## Description

`hesschek(net, x, t)` takes a network data structure `net`, together
with input and target data matrices `x` and `t`, and compares
the evaluation of the Hessian matrix using the function `nethess`
and using central differences with the function `neterr`.

The optional return value `h` is the Hessian computed using
`nethess`.

## See Also

`nethess`, `neterr`

---

**Pages:**
Index


---

Copyright (c) Ian T Nabney (1996-9)
